# A Herbal Mixture Formula of OCD20015-V009 Prophylactic Administration to Enhance Interferon-Mediated Antiviral Activity Against Influenza A Virus

**DOI:** 10.3389/fphar.2021.764297

**Published:** 2021-11-24

**Authors:** Eun-Bin Kwon, You-Chang Oh, Youn-Hwan Hwang, Wei Li, Seok-Man Park, Ryong Kong, Young Soo Kim, Jang-Gi Choi

**Affiliations:** ^1^ Korean Medicine (KM) Application Center, Korea Institute of Oriental Medicine (KIOM), Daegu, South Korea; ^2^ Herbal Medicine Research Division, Korea Institute of Oriental Medicine, Daejeon, South Korea; ^3^ Okchungdang, Daegu, South Korea

**Keywords:** OCD20015-V009, influenza A virus, antiviral activity, interferon, herbal complex medicine

## Abstract

OCD20015-V009 is an herbal mix of water-extracted Ginseng Radix, Poria (Hoelen), Rehmanniae Radix, Adenophorae Radix, Platycodi Radix, Crataegii Fructus, and Astragali Radix. In this study, its *in vitro* and *in vivo* antiviral activity and mechanisms against the influenza A virus were evaluated using a GFP-tagged influenza A virus (A/PR/8/34-GFP) to infect murine macrophages. We found that OCD20015-V009 pre-treatment substantially reduced A/PR/8/34-GFP replication. Also, OCD20015-V009 pre-treatment increased the phosphorylation of type-I IFN-related proteins TBK-1 and STAT1 and the secretion of pro-inflammatory cytokines TNF-α and IL-6 by murine macrophages. Moreover, OCD20015-V009 prophylactic administration increased IFN-stimulated genes-related 15, 20, and 56 and IFN-β mRNA *in vitro*. Thus, OCD20015-V009 likely modulates murine innate immune response *via* macrophages. This finding is potentially useful for developing prophylactics or therapeutics against the influenza A virus. Furthermore, pre-treatment with OCD20015-V009 decreased the mortality of the mice exposed to A/PR/8/34-GFP by 20% compared to that in the untreated animals. Thus, OCD20015-V009 stimulates the antiviral response in murine macrophages and mice to viral infections. Additionally, we identified chlorogenic acid and ginsenoside Rd as the antiviral components in OCD20015-V009. Further investigations are needed to elucidate the protective effects of active components of OCD20015-V009 against influenza A viruses.

## Introduction

The genome of the influenza A virus (IAV) contains eight segments of negative-sense single-stranded RNA and remains a major threat to public health. IAV infection leads to enormous morbidity and economic loss (Lam et al., 2013); each year, seasonal influenza virus (IV) infects 5–15% of the global human population causing approximately 300,000–500,000 deaths (Clayville, 2011; Kim et al., 2020). Several epidemics and pandemics have occurred over the past century due to antigenic drift or shift in the IAV (Yin et al., 2018; Degoot et al., 2019). Antigenic shifts are substantial in the influenza A virus caused by genetic re-assortment, resulting in novel hemagglutinin (HA) and/or novel HA and neuraminidase (NA) from Avian Influenza into currently circulating human influenza viruses that infect humans (Yin et al., 2018; Degoot et al., 2019). However, predicting the next antigen shift or the resultant outbreak is challenging (Yin et al., 2018; Degoot et al., 2019). Moreover, influenza vaccines have become ineffective (Berlanda Scorza et al., 2016). Thus, antiviral agents are crucial in disease control (Berlanda Scorza et al., 2016; Pardi and Weissman, 2020). Anti-influenza medications such as matrix protein 2 (M2) ion channel blockers (Takeda et al., 2002) and neuraminidase (NA) inhibitors (Alymova et al., 2005) have been approved globally, while an RNA polymerase inhibitor (Hayden and Shindo, 2019), an antivirus medication, has been approved regionally (Zhang et al., 2019). Additionally, NA inhibitors such as oseltamivir and zanamivir are frequently administered. However, due to the emergence of resistant influenza strains, M2 ion channel blockers, such as amantadine and rimantadine, are rarely used. Furthermore, the resistance of influenza strains to NA inhibitors, such as oseltamivir and zanamivir, has increased (Hussain et al., 2017).

Conversely, the host initiates the innate immune system, the first line of defense against most pathogens, including the influenza virus, *via* the production of antiviral cytokines (Chen et al., 2018). For instance, activated interferon (IFN) is a critical component of innate immunity against the influenza virus (Chen et al., 2018; Wu and Metcalf, 2020). Type I IFN plays a crucial role in the innate immune response against many viruses and is also a component of the adaptive immune response to viral and nonviral pathogens (Chen et al., 2018; Wu and Metcalf, 2020). However, overproduction of IFNs and proinflammatory factors may cause a cytokine storm that aggravates a disease by disrupting the immune suppression of viral infections and causing tissue damage. Thus, IFN and proinflammatory cytokines, the first line of defense against the influenza virus, act as a double-edged sword (Gu et al., 2019).

After the influenza virus infects the lungs, type I IFNs are rapidly expressed in numerous myeloid and parenchymal cells (Jewell et al., 2007; Kumagai et al., 2007). Type I IFNs’ pro-inflammatory activity allows for immunological modulation of this antiviral cytokine family (Kopitar-Jerala, 2017). Cytokine secretion from macrophages is tightly regulated at the transcriptional level. Post-transcriptional modulation of IFNs and proinflammatory cytokines also occurs (Kopitar-Jerala, 2017). The innate immune response is involved in the various inflammatory processes and is particularly vital for viral infections, which affect the cellular, tissue, and overall physiological functions (Teijaro, 2016; Kopitar-Jerala, 2017). Therefore, rapid IFN production is required during viral infection to inhibit virus spread in the host cells (Teijaro, 2016; Kopitar-Jerala, 2017).

Traditional herbal medicines or natural products such as Clove (*Syzygium aromaticum* (L.) Merr. & L.M. Perry) and *Opuntia ficus-indica* (L.) strengthen the antiviral properties against influenza or SARS-CoV-2 (Vicidomini et al., 2021a; Vicidomini et al., 2021b). Particularly, IFN-β and proinflammatory cytokines are essential in the defense against the IAV (Koerner et al., 2007). The activity of IFN-β and proinflammatory cytokines can be enhanced with traditional herbal medicines or herbal products that strengthen the host’s antiviral defense response to influenza (Talactac et al., 2015; Mousa, 2017; Trinh et al., 2020). Therefore, researchers are examining herbal medications or natural compounds with immunomodulatory properties for influenza virus infection treatment (Choi et al., 2016; Choi et al., 2017a; Choi et al., 2017b). In this study, we investigated whether OCD20015-V009-induced signaling triggers antiviral mediators, such as type I interferons, proinflammatory cytokines, and interferon-stimulatory genes responsible for the antiviral state in murine macrophage cells. Here, we investigated whether OCD20015-V009, a herbal complex containing the water extract of *Panax ginseng* C.A.Mey. (Ginseng Radix), *Wolfiporia extensa* (Peck) Ginns (syn. Poria cocos (Schw.)) (Poria or Hoelen), *Rehmannia glutinosa* (Gaertn.) DC. (Rehmanniae Radix), *Adenophora triphylla* (Thunb.) A.DC. (Adenophorae Radix), *Platycodon grandiflorus* (Jacq.) A.DC. (Platycodi Radix), *Crataegus pinnatifida* Bunge (Crataegii Fructus), and *Astragalus mongholicus* Bunge (Astragali Radix), could inhibit influenza virus infection *in vitro* and *in vivo*. We first examined OCD20015-V009s potential in impeding the influenza virus infection *in vitro*. Then, we investigated whether OCD20015-V009 could protect mice from a lethal challenge with an H1N1 subtype of IAV.

## Materials and Methods

### OCD20015-V009 Preparation

A total of 2,000 g of dried OCD20015-V009, including *Panax ginseng* C.A.Mey. (Ginseng Radix) (50 g), *Wolfiporia extensa* (Peck) Ginns (syn. Poria cocos (Schw.)) (Poria or Hoelen) (100 g), *Rehmannia glutinosa* (Gaertn.) DC. (Rehmanniae Radix) (500 g), *Adenophora triphylla* (Thunb.) A.DC. (Adenophorae Radix) (50 g), *Platycodon grandiflorus* (Jacq.) A.DC. (Platycodi Radix) (275 g), *Crataegus pinnatifida* Bunge (Crataegii Fructus) (25g), and *Astragalus mongholicus* Bunge (Astragali Radix) (1,000 g) (Okchundang, Daegu, Korea), was prepared by immersion in 10 L of distilled water and heat-extraction at 115°C for 3 h. After filtration through a 150-µm sieve, OCD20015-V009 was freeze-dried. The yield of the OCD20015-V009 extract was 14.1% (283.7 g); it was stored in the KM-Application Center herbarium (registration number, #OCD 2020-1) Korea Institute of Oriental Medicine (KIOM) in desiccators at 4°C until further use.

### UHPLC-MS/MS Analysis

Ajugol, calycosin, calycosin-7-glucoside, catalpol, chlorogenic acid, epicatechin, formononetin, ginsenoside Rb1, ginsenoside Rc, ginsenoside Rd, ginsenoside Re, ginsenoside Rf, ginsenoside Rg1, ginsenoside Rg5, ginsenoside Rk2, ginsenoside Ro, isoquercitrin, loganic acid, and notoginsenoside R2 (Targetmol, United States) were purchased. 3-Epidehydrotumulosic acid, 6α-hydroxypolyporenic acid C, dehydropachymic acid, dehydrotumulosic acid, pachymic acid, platycodin D, platycodin D2, polyporenic acid C, and poricoic acid A (ChemFaces, China) were purchased from ChemFaces (Wuhan, Hubei, China). A Dionex UltiMate 3000 system equipped with a Thermo Q-Exactive mass spectrometer (UHPLC-MS/MS, Thermo Fisher Scientific, United States) was used for the phytochemical analysis of OCD20015-V009. Data acquisition and processing were performed using Xcalibur v.3.0 and Tracefinder v.4.0. Chromatographic separation was achieved with an Acquity BEH C18 column (100 mm × 2.1 mm, 1.7 μm, Waters, United States) with gradient elution consisting of 0.1% formic acid in water and acetonitrile (Hwang et al., 2019; Hwang et al., 2020). The identified compounds were compared to the retention time and mass spectrum of the authenticated standards. For the constituents that did not match the standards, we found corresponding m/z and the MS fragment information from previous reports (Lee et al., 2019; Santoro et al., 2020).

### Cell Lines and Virus

RAW 264.7 (murine macrophage) and Madin-Darby canine kidney (MDCK, NBL-2) cells (American Type Culture Collection) were cultured at 37°C in a 5% CO_2_ incubator in Dulbecco’s modified eagle medium (DMEM; Lonza, United States) containing 10% fetal bovine serum (FBS; Biotechnics Research, United States) and 1% penicillin and streptomycin (Cellgro, United States). Influenza A (A/Puerto Rico/8/34 (A/PR/8/34) from American Type Culture Collection (ATCC, VR-95™) and GFP-tagged A/PR/8/34 virus (A/PR/8/34-GFP) used in previous studies (Choi et al., 2019). A/PR/8/34-GFP was briefly constructed by fusing the GFP gene to the C-terminal end of the nonstructural protein 1 open reading frame (NS1 ORF), which had a silent mutation at the splice acceptor without the stop codon, followed by an autoproteolytic site and nuclear export protein (Choi et al., 2019).

Accoding to a prior publication, the replication and viral titer of the two strains were determined (Choi et al., 2019).

### Reagents and Antibodies

Recombinant mouse IFN-β (Sigma-Aldrich, United States), antibodies against influenza virus proteins M1, NA, NP and PA (GeneTex, United States), antibodies against cellular proteins tubulin, p-STAT1, STAT1, p-TBK1 and TBK1 (Cell Signaling Technology, United States), horseradish peroxidase (HRP)-conjugated secondary antibodies (Cell Signaling Technology), DMEM, FBS, antibiotics (Hyclone, United States), lipopolysaccharide (LPS), bovine serum albumin (BSA) (Sigma-Aldrich), and 100 mm culture dishes and 6 or 96-well plates (Sarstedt, Germany) were purchased. Enzyme-linked immunosorbent assay (ELISA) antibody sets (eBioscience, United States), and RNA extraction kit (iNtRON Biotech, Korea), oligonucleotide primers for quantitative real-time polymerase chain reaction (qRT-PCR), DNA synthesizing kits, and the AccuPower® 2× Greenstar qPCR Master Mix (Bioneer, Korea) were procured.

### Cell Viability Assay

Cell viability was determined using the MTT assay. The RAW 264.7 and MDCK cells were seeded into 24-well plates at 1 × 10^5^ cells/well, and OCD20015-V009 was added to the wells at a concentration of 0–400 μg/ml. MTT solutions were added to each well after 24 h, and the cells were incubated for another 30 min (D'Alessandro et al., 2019; Mosmann, 1983). Subsequently, 1 ml DMSO was added before measuring the absorbance at 540 nm using an Epoch Microplate Reader (BioTek, United States). The data were represented by the mean ± SEM of four independent experiments.

### Cytokine Determination

For ELISA, RAW 264.7 cells were seeded and incubated for 18 h. The cells were treated with 20 ng/ml LPS or OCD20015-V009 at 50 or 100 μg/ml for 6 or 24 h. The levels of the inflammatory cytokine TNF-α and IL-6 in the culture medium were measured using the ELISA antibody set purchased from eBioscience (#88-7324-77 and #88-7064-77).

### Total RNA Extraction and qRT-PCR

Total RNA extraction and cDNA synthesis were conducted using the Easy-BLUE™ RNA extraction kits (iNtRON Biotech) and AccuPower® CycleScript RT PreMix (Bioneer), respectively. A total of 1 μg RNA was reverse-transcribed into cDNA, and qPCR oligonucleotide primers for macrophage cell cDNA are indicated in Table 1 qPCR reactions were performed in triplicate 20 μL reactions with 1 μL of 0.3 μM of the forward and reverse primer each, 10 μL of the AccuPower® 2× Greenstar qPCR master mix, 5 μL of template DNA, and 3 μL of RNase-free water. The PCR cycle was as follows: 95°C for 10 min, 40 cycles of 95°C for 20 s, 60°C (ISG15), 53°C (ISG20), 60°C (IFN-β), 56°C (ISG56), or 60°C (TNF-α) for 40 s, and at each experiment end, a melting curve analysis was conducted to confirm that a single product per primer pair was amplified. Amplification and analysis were performed using the QuantStudio 6 Flex Real-time PCR System (Thermo Fisher), and each sample was compared using the relative CT method. Fold changes in gene expression were determined relative to the blank control after normalization to GAPDH expression using the 2−ΔΔCt method.

**TABLE 1:** Primers sequences for real-time RT-PCR.

Name	Orientation	Primer sequences
		5–3′ orientation
GAPDH	Forward	TGA​CCA​CAG​TCC​ATG​CCA​TC
	Reverse	GAC​GGA​CAC​ATT​GGG​GGT​AG
ISG-15	Forward	CAA​TGG​CCT​GGG​ACC​TAA​A
	Reverse	CTT​CTT​CAG​TTC​TGA​CAC​CGT​CAT
ISG-20	Forward	AGA​GAT​CAC​GGA​CTA​CAG​AA
	Reverse	TCT​GTG​GAC​GTG​TCA​TAG​AT
ISG-56	Forward	AGA​GAA​CAG​CTA​CCA​CCT​TT
	Reverse	TGG​ACC​TGC​TCT​GAG​ATT​CT
TNF- α	Forward	AGC​AAA​CCA​CCA​AGT​GGA​GGA
	Reverse	GCT​GGC​ACC​ACT​AGT​TGG​TTG​T
IFN- β	Forward	TCC​AAG​AAA​GGA​CGA​ACA​TTC​G
	Reverse	TGC​GGA​CAT​CTC​CCA​CGT​CAA

### Viral Replication Inhibition Assay

A viral replication inhibition assay was performed using the A/PR/8/34 and A/PR/8/34-GFP viruses (Choi et al., 2019). We tested the antiviral effect of OCD20015-V009 on viruses previously used for virus challenge studies, such as A/PR/8/34-GFP (Choi et al., 2019). RAW 264.7 cells were seeded in 24-well plates at 1 × 10^5^ cells/well and incubated for 24 h. The cells were incubated for 18 h in DMEM alone (for the untreated or virus-only group), DMEM with 1,000 U of recombinant mouse interferon (IFN-β, as the positive control), or DMEM with 50 and 100 μg/ml OCD20015-V009. The cells were then infected with A/PR/8/34-GFP at a multiplicity of infection (MOI) of 10. The GFP levels were measured at 24 h post-infection (hpi) at 200 × magnification under a fluorescence microscope (Nikon, Japan). After, cells were harvested using trypsinization followed by fluorescence detection using flow cytometry (CytoFLEX, Beckman, United States) (Choi et al., 2019).

### Plaque Assay

Raw264.7 cells were cultured in 24-well plates (1 × 10^5^ cells/ml) for 24 h. Then, various concentrations of OCD2015-V009 were added and incubated at 37°C for 18 h. Following the reaction, cells were infected with H1N1 for 2 h, rinsed with phosphate-buffered saline (PBS), and complete DMEM was added to the medium for 24 h. Then, MDCK cells were infected for 2 h with the Raw264.7 cell culture supernatant containing viruses. After that, MDCK monolayers were coated with 1.5% agarose in 2X complete DMEM and incubated with 5% CO_2_ at 37°C for 3 days. Cells were stained with 1% crystal violet solution following incubation or infection, and plaques were enumberated.

### Immunofluorescence Staining

RAW 264.7 cells seeded onto cover slides at 1 × 10^5^ cells/ml were cultured at 37°C with 5% CO_2_ for 24 h. The cells were then pre-treated with OCD20015-V009 or IFN-β and incubated at 37°C with 5% CO_2_ for 18 h before infection with A/PuertoRico/8/34 at the MOI of 10 for 2 h. After viral infection, the virus and medium were removed, and the cells were rinsed with phosphate-buffered saline (PBS) thrice. Next, a complete medium was added, and the cells were incubated at 37°C with 5% CO_2_. After 24 h, the cells were rinsed with cold PBS, fixed with 4% paraformaldehyde for 30 min at room temperature, and permeabilized with 0.1% Triton-X100 in PBS for 15 min. After blocking, the cells were incubated with a rabbit polyclonal antibody against M2 (1:250 in 3% BSA; GeneTex, United States) at 4°C overnight, rinsed with cold PBS thrice, and incubated with an Alexa Fluor 568 goat anti-rabbit IgG antibody (1:500 in 3% BSA; Thermo Fisher) for 1 h. The nuclei were visualized by staining with DAPI (0.5 μg/ml; Thermo Fisher) for 10 min. Then, the images were captured using a fluorescence microscope (Nikon).

### Western Blot

The RAW 264.7 cells seeded in 6-well plates at 1 × 10^6^ cells/well were incubated with OCD20015-V009 and LPS at 37°C with 5% CO_2_. Afterward, the cells were harvested and lysed in RIPA buffer (Millipore, United States) containing protease and phosphatase inhibitors. The total protein content in the samples was normalized using Bradford’s reagents. The proteins were separated using SDS-PAGE and transferred to a polyvinylidene fluoride membrane (Millipore). After blocking with 3% BSA, the blots were incubated with primary anti-STAT1, anti-TBK1, anti-phospho-STAT1, anti-phospho-TBK1, anti-β-actin, M1, NA, NP, and PA antibodies (1:1,000 dilution) at 4°C overnight. After the blots were washed in TBS-T thrice, they were incubated with an HRP-conjugated secondary antibody. The proteins were quantified using a ChemiDoc™ Touch Imaging System (Bio-Rad), and the relative intensities of protein bands were measured using ImageJ.

### Animal Studies

This study was conducted following the guidelines of the Institutional Animal Care and Use Committee (IACUC) of the Laboratory Animal Center (LAC) of Daegu-Gyeongbuk Medical Innovation Foundation (DGMIF). The animal studies were approved by the IACUC of the LAC of DGMIF under approval number DGMIF-18071602-00.

Five-week-old female BALB/c mice (Orient Bio Inc., Seongnam, South Korea) were acclimated for at least 1 week under standard housing conditions at the LAC of DGMIF and provided with a standard rodent chow diet and water ad libitum. For the oral inoculation of OCD20015-V009 and the IAV challenge, the mice were separated into four experimental groups of ten mice in each group and administered with control, PBS, 100 or 300 mg/kg OCD20015-V009 with IAV, respectively. The group of mice without virus infection was used as a negative control. The mice in each experimental group were orally administered PBS, 100 and 300 mg/kg OCD20015-V009 at a total volume of 200 µL once daily for 7 days before infection, respectively (Choi et al., 2017a). The mice were infected intranasally with 20 µL of A/PR/8/34 in PBS at the 50% mouse lethal dose (LD50).

Survival was monitored for 10 days post-infection (dpi) at fixed time points. At 7 dpi, three mice from each group were randomly selected and sacrificed to measure lung histopathology. The lung tissue samples were immediately fixed in paraffin-embedded neutral buffer containing 10% formalin and sliced to 4–6-µm sections using a microtome. The sections were mounted on a slide, stained with eosin, and examined under an optical microscope (Choi et al., 2017a). The remaining mice were used to measure survival at 10 dpi.

### Statistical Analysis

The data were expressed as mean ± SEM. The significance of the differences in the mean values between the treatment and control groups was determined using one-way ANOVA. Additionally, Tukey’s post-hoc test was utilized for multi-group comparisons. Analyses were performed using GraphPad PRISM® Version 5.02 (GraphPad, United States). A p-value less than 0.05 denotes statistical significance.

## Results and Discussion

### Effects of OCD20015-V009 on Cell Cytotoxicity

**FIGURE 1:**
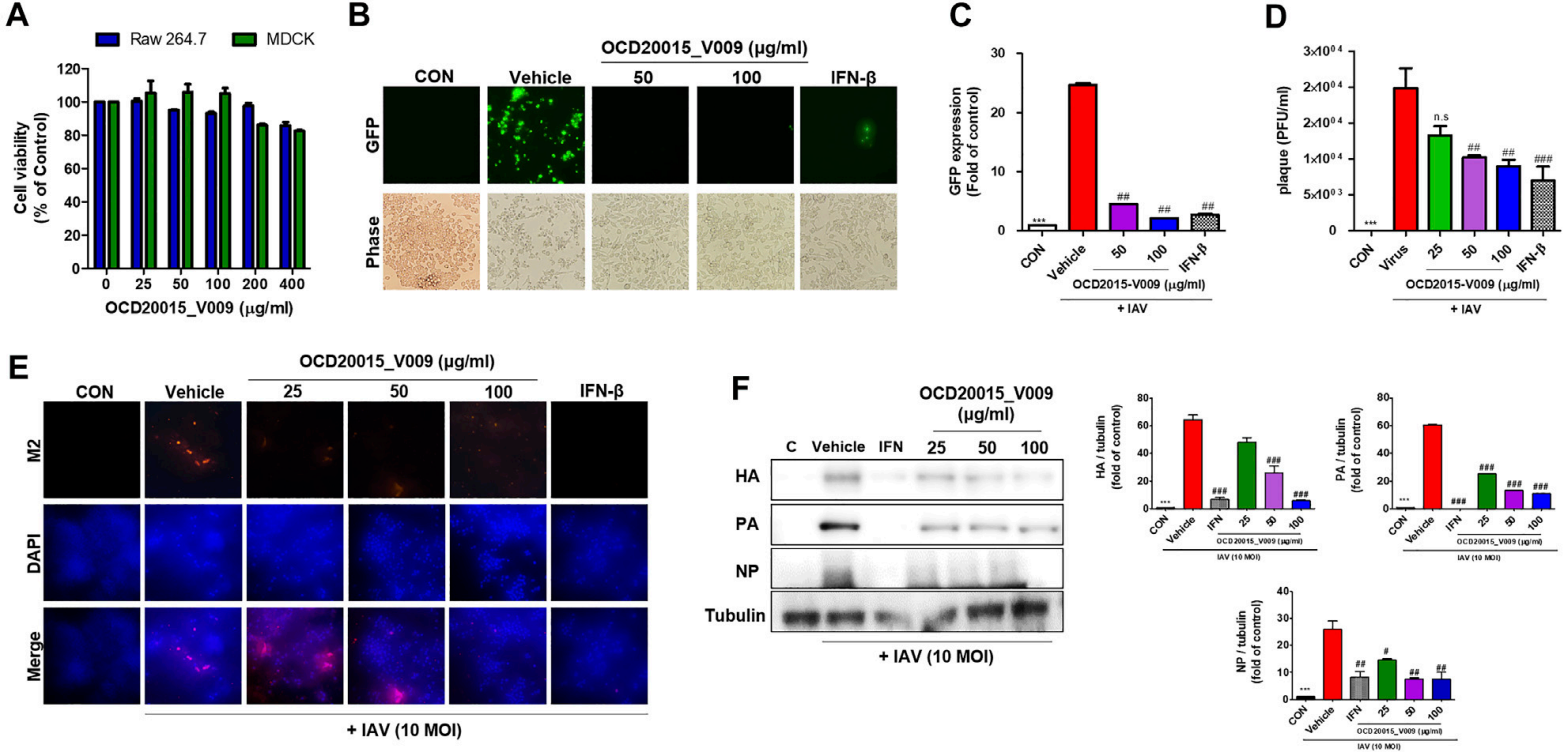
The antiviral effect of OCD20015-V009 pre-treatment on the influenza A virus infection of RAW 264.7 murine macrophages. **(A)** Determination of the effective cytotoxic concentration of the OCD20015-V009 in RAW 264.7 and MDCK cells. Cell viability after 24 h (*n* = 3 each) was determined using the MTT assay. **(B)** RAW 264.7 cells were treated with OCD20015-V009 before IAV infection. The cells were incubated with only a medium, 50 and 100 μg/ml OCD20015-V009, or 1000 U/mL recombinant mouse interferon-β for 12 h before infection with A/PR/8/34-GFP at a multiplicity of infection of 10. Images of GFP production were obtained 24 h after virus infection. **(C)** The GFP levels and reduction in viral replication were assessed using flow cytometry 24 h after viral infection in the OCD20015-V009-treated RAW 264.7 cells. The data are presented as the mean ± SD (error bars) of three independent experiments. ***p < 0.001 compared with the control, ##*p* < 0.01 compared with the vehicle (IAV-infection) **(D)** Effects of OCD2015-V009 treatment on A/PR/8/34 infection and viral growth in plaques in MDCK cells. **(E)** The reduction of M2 in RAW 264.7 cells was observed with fluorescence microscopy using an M2-specific antibody. RAW 264.7 cells were stained with DAPI (blue), and the merged images represent M2 (red). RAW 264.7 cells were pre-treated with 25, 50 or 100 μg/ml OCD20015-V009, 1000 U/mL recombinant mouse interferon (IFN)-β, or only the medium (negative control) before viral adsorption. **(F)** The levels of influenza A virus proteins HA, PA, and NP in cell lysates were analyzed by Western blots, and the level of tubulin was used as internal control. Western blotting of viral expressions followed by quantification using the ImageJ software was performed. The data are presented as the mean ± SD (error bars) of three independent experiments. **p < 0.01 indicates a significant difference between control groups, #*p* < 0.05 and ##*p* < 0.01 indicates a significant difference between the vehicle (IAV-infection) groups. n.s., not significant, compared with the pre-treatment.

The cytotoxicity of OCD20015-V009 was investigated by incubating RAW 264.7 cells with OCD20015-V009 at 0–400 μg/ml for 24 h. OCD20015-V009 did not exhibit cytotoxicity in the RAW 264.7 cells (Figure 1A). Therefore, subsequent experiments were conducted with OCD20015-V009 below 100 or 200 μg/ml.

### OCD20015-V009 Inhibited IAV Infection in RAW 264.7 Cells

The antiviral activity of OCD20015-V009 was examined by detecting GFP levels in RAW 264.7 cells after suppressed A/PR/8/34-GFP replication. The untreated cells had high GFP levels upon infection by A/PR/8/34-GFP. Conversely, the GFP level of RAW 264.7 cells pre-treated with OCD20015-V009 was considerably lower (Figure 1B). The replication of A/PR/8/34-GFP in RAW 264.7 cells was significantly decreased by 81.5 and 91.1% with OCD20015-V009 pre-treatment at 50 and 100 μg/ml, respectively, compared to the vehicle (the virus treatment) group (Figure 1C). Furthermore, we observed that dose-dependent OCD20015-V009 decreased plaque formation in MDCK cells (Figure 1D).

Immunofluorescence (IF) and Western blots were performed to validate the production of IAV proteins. Cells pre-treated with OCD20015-V009 produced significantly less M2 (Figure 1E). Additionally, the production of HA, PA, and NP was significantly inhibited in RAW 264.7 cells pre-treated with 100 μg/ml OCD20015-V009 before infection with A/PR/8/34(H1N1)-GFP (Figure 1F).

These data imply that OCD20015-V009 pre-treatment significantly inhibits IAV infection and viral protein production in RAW 264.7 cells. Thus, OCD20015-V009 pre-treatment likely reduces influenza H1N1 viral protein production and inhibits infection.

### OCD20015-V009 Inhibited IAV Infection *in vivo*

**FIGURE 2:**
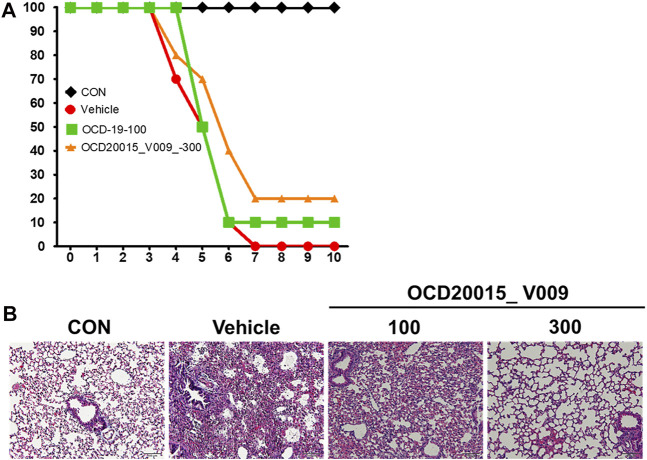
The effect of OCD20015-V009 pre-treatment on influenza A virus infection in mice. The BALB/c mice were pre-treated orally with 100 or 300 mg/kg OCD20015-V009 (200 μL/mouse) 7 days before viral infection. **(A)** The daily percentage of survival until 10 days post-infection. **(B)** A representative H&E image of the histopathological damage in sectioned lung tissue samples from the untreated mice or mice pre-treated with OCD20015-V009.

We investigated the protective effects of OCD20015-V009 on IAV infection in BALB/c mice. The mice treated once daily with 100 or 300 mg/kg OCD20015-V009 maintained a relatively stable body weight with no significant clinical symptoms in this study (data not shown). All untreated A/PR/8/34-infected mice were dead within 7 dpi (Figure 2). Contrarily, the mortality of the mice pre-treated with OCD20015-V009 after A/PR/8/34 infection was reduced (Figure 2A). The lungs from the mice were sampled at 7 dpi for hematoxylin and eosin staining to investigate histopathological changes caused by viral infection. Lung inflammation or pathological changes were not found in the normal control group (Figure 2B). However, bronchial epithelial cells were necrotized with thickened alveolar walls in the mice in the vehicle group; in addition, severe pulmonary congestion and lesions were observed. Also, the alveolar space was occupied with moderate inflammatory infiltrates of neutrophils, macrophages, and lymphocytes. However, lung samples from the mice pre-treated with 300 mg/kg OCD20015-V009 revealed pulmonary congestion and lesion alleviation, indicating lower lung inflammation compared to untreated mice (Figure 2B).

### Effects of OCD20015-V009 on Pro-Inflammatory Cytokine Production and Type-I IFN Signaling Pathway Activation in RAW 264.7 Murine Macrophages

Pro-inflammatory cytokines and type-I IFN are significant in inducing immunoregulatory activities and antiviral responses. Herbal medicine’s immunomodulatory effect for treating IAV infection has been extensively studied (Choi et al., 2017a). Additionally, innate immune responses through the production of pro-inflammatory cytokines and type-I IFN may be responsible for OCD20015-V009s antiviral action. Using ELISA, we evaluated the effect of OCD20015-V009 on TNF-α and IL-6 the secretion.

**FIGURE 3:**
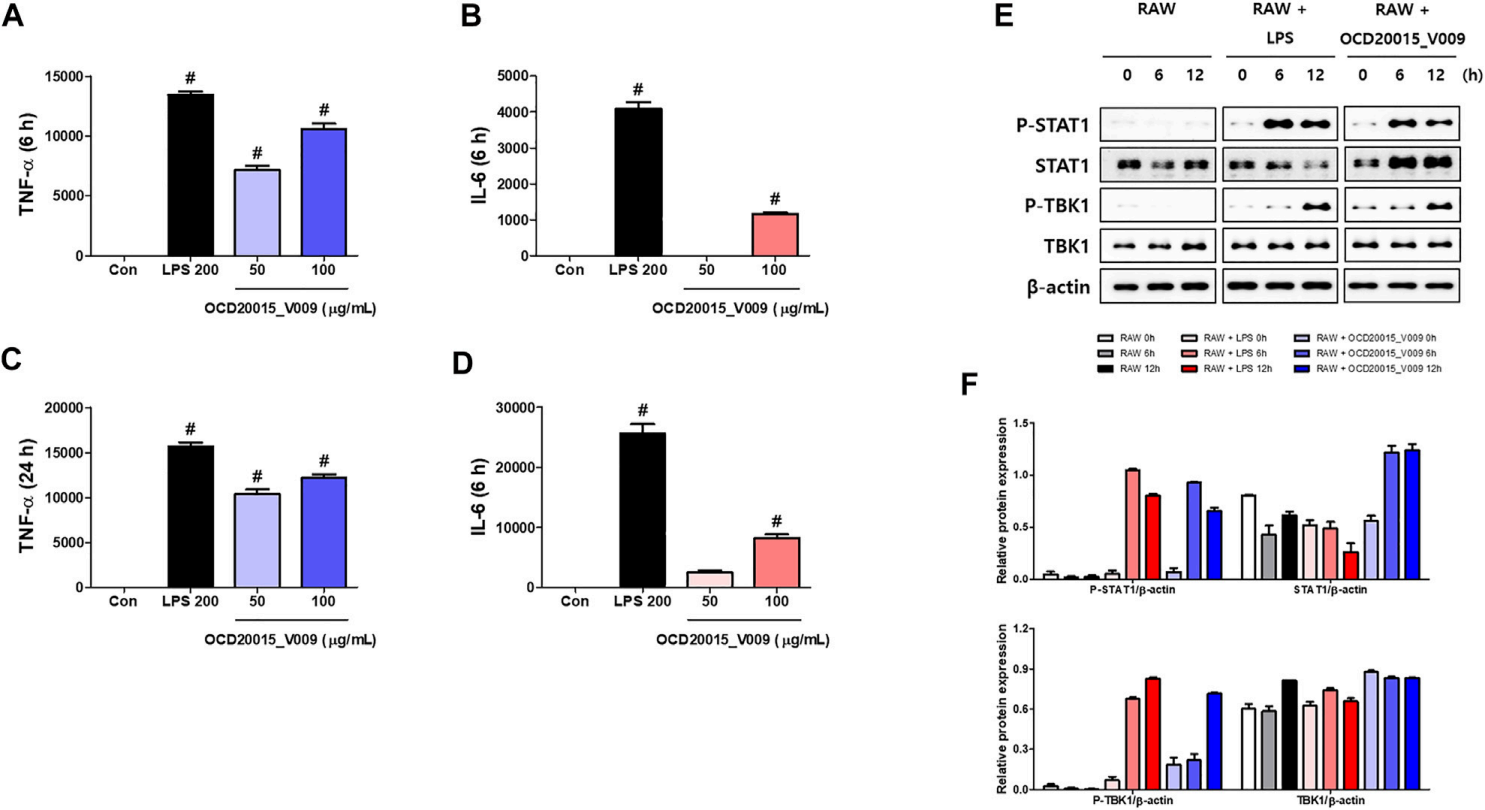
The activation of type-I interferon (IFN) and induction of pro-inflammatory cytokines by OCD20015-V009 in RAW 264.7 cells. **(A–D)** The cells were treated with vehicle alone, 200 ng/ml LPS, or 50 and 100 μg/ml OCD20015-V009 and incubated at 37°C. The supernatant from each treatment group was harvested at 6 or 24 h and centrifuged at 15,000 g for 10 min at 4°C. The clarified supernatants were dispensed into the enzyme-linked immunosorbent assay plates coated with the captured antibody of murine interleukin (IL)-6 or tumor necrosis factor (TNF)-α to measure cytokine secretion. The experiment was performed in triplicate. **(E,F)** Western blotting of whole-cell lysates of macrophages treated with vehicle alone, 200 ng/ml LPS, or 200 μg/ml OCD20015-V009 was performed to assess the level of the non-phosphorylated and phosphorylated forms of TANK-binding kinase 1 (TBK1), STAT1, and β-actin over time. **p < 0.01 and ***p < 0.001 indicate a significant difference between control groups.

The concentrations of secreted TNF-α and IL-6 increased by 10,597.4 ± 768.9 and 1165.5 ± 95.9 compared to the control when 100 μg/ml OCD20015-V009 was treated for 6 h, respectively, and after 24 h, 12,155.13 ± 667 and 8250.2 ± 975.2 increased compared to control. (Figures 3A–D). (Figures 3A–D). These results indicate that OCD20015-V009 induces the antiviral response mediated by TNF-α and IL-6 in murine macrophages.

Additionally, we studied TBK1 and STAT1 phosphorylation in RAW 264.7 cells pre-treated with OCD20015-V009 using Western blots to determine the OCD20015-V009 effect on the activation of type-I IFN signaling molecules. The results show that OCD20015-V009 treatment upregulates STAT1 and TBK1 phosphorylation, and they are key molecules in the type-I IFN signaling pathway (Figures 3E,F).

**FIGURE 4:**
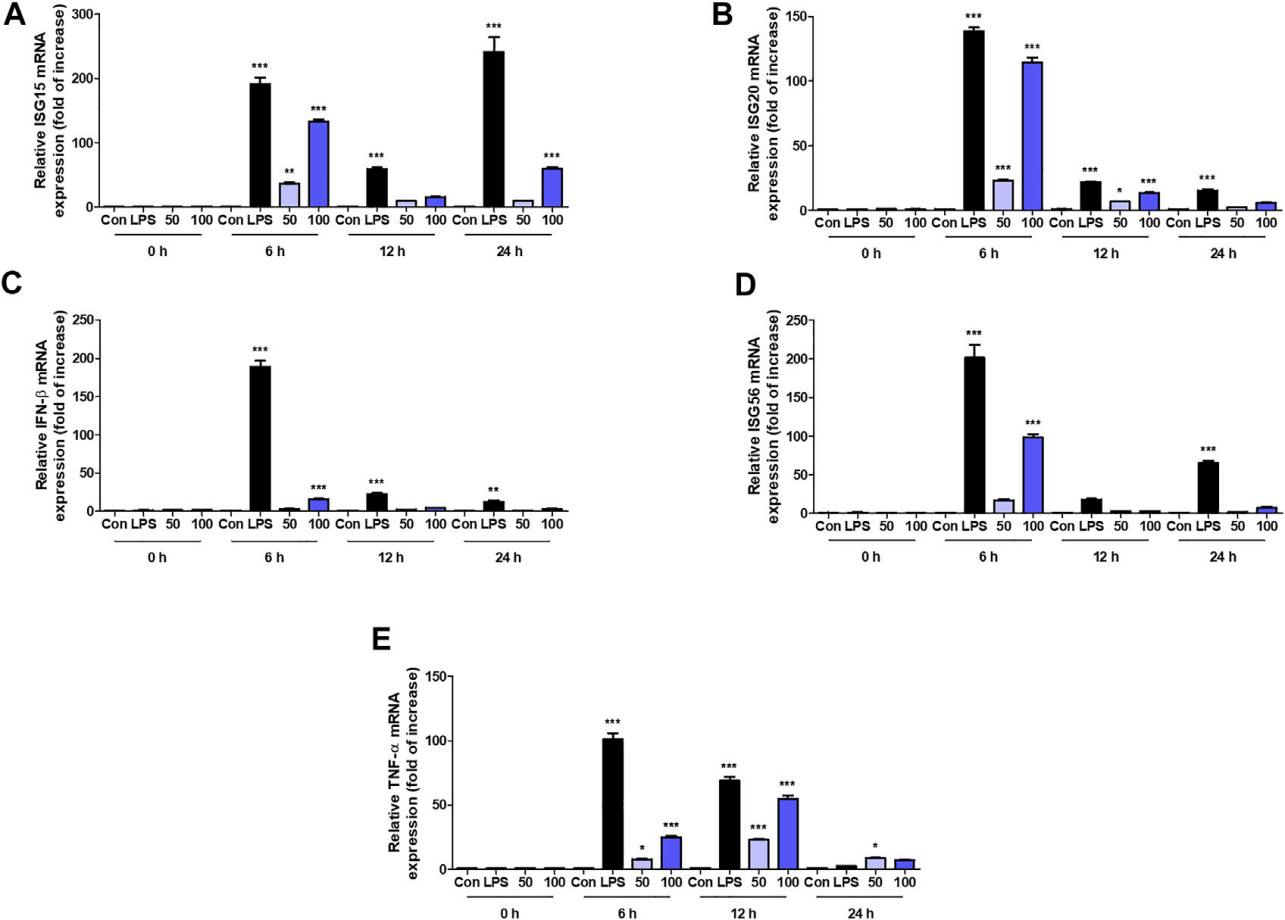
Induction of transcription of the interferon (IFN)-related gene and IFN-stimulated genes (ISGs) by OCD20015-V009 in RAW 264.7 cells. The cells were treated with the vehicle alone (Con), 200 ng/ml lipopolysaccharides (LPS), or 50 or 100 μg/ml OCD20015-V009 and then incubated at 37°C with 5% CO_2_. The time-dependent changes in the mRNA levels of ISG-15, 20, and 56, TNF-α, and IFN-β genes **(A–E)** in RAW 264.7 cells after OCD20015-V009 treatment were examined. The data are representative of three independent experiments. **p < 0.01 and ***p < 0.001 indicates a significant difference between control groups.

We further analyzed the interaction between OCD20015-V009 and IFN-stimulated genes in RAW 264.7 cells. The expression of the IFN-stimulated gene (ISG)-15, ISG-20, and ISG-56, and TNF-α and IFN-β genes time-dependently increased in the OCD20015-V009-treated RAW 264.7 cells compared with that in the untreated cells (Figures 4A–E). Additionally, the upregulation of the TNF-α, IFN-β, and ISG was notable. The observed pattern was similar to that of LPS-treated positive control (Figure 4); the transcription of the ISG-15, ISG-20, and ISG-56 genes increased by 132.9 ± 5.2, 114.2 ± 6.7, and 98.1 ± 7.5-fold, respectively, in the cells pre-treated with 100 μg/ml OCD20015-V009 for 6 h (Figures 4A–E). Overall, the results indicate that OCD20015-V009 can induce an antiviral state by modulating the IFN signaling pathway and ISG expression in macrophages, thus inhibiting viral infection.

### Chemical Composition of OCD20015-V009 by UPLC-MS/MS Analysis

**FIGURE 5:**
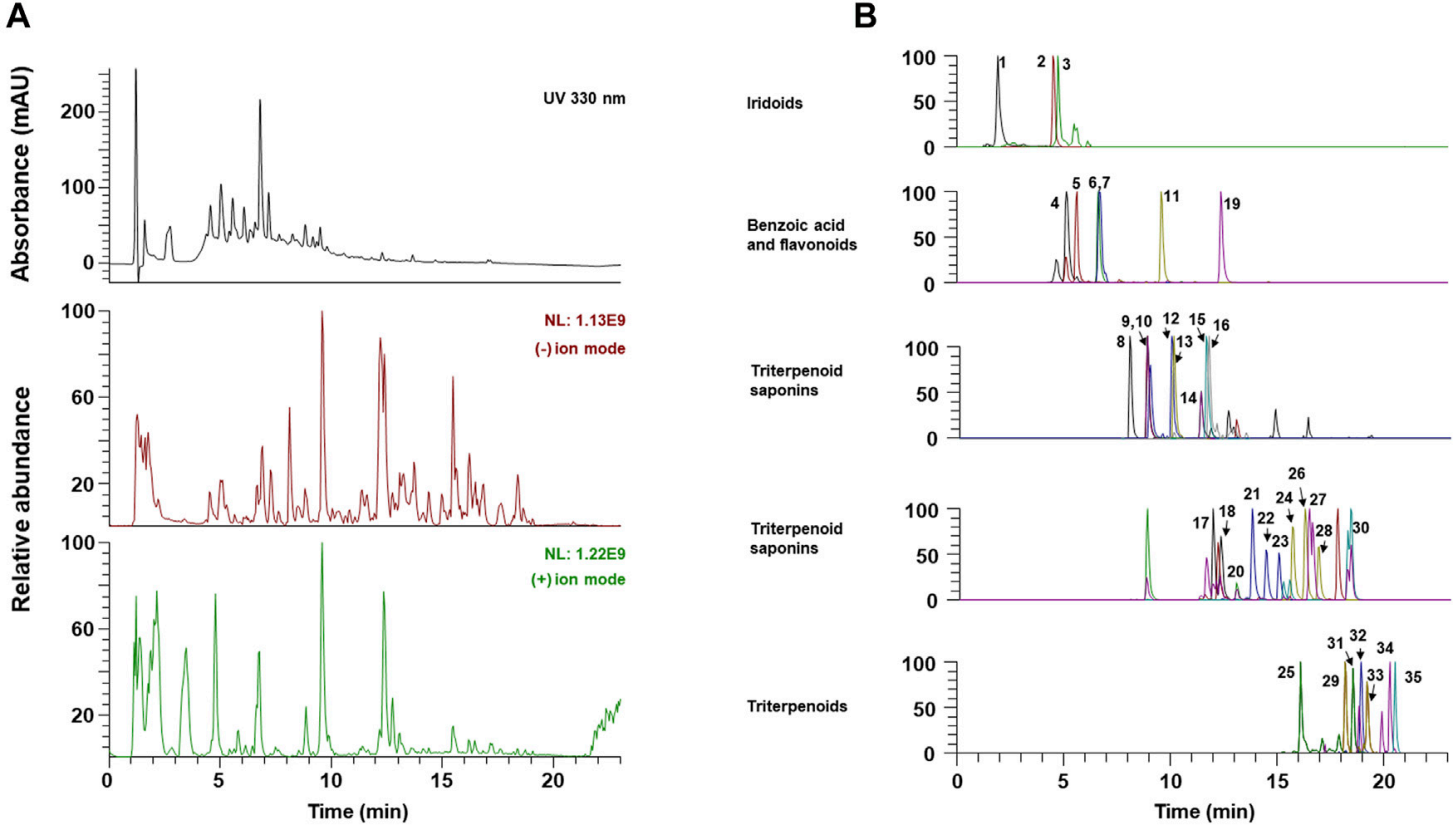
The UPLC-MS/MS chromatograms of OCD20015-V009 identified phytochemicals. **(A)** UV chromatogram and total ion chromatogram of OCD20015-V009. **(B)** The PRM chromatogram of OCD20015-V009.

The components of OCD20015-V009 were identified by analyzing the water extracts of each traditionally used herb. The UPLC-MS/MS analysis, which compared the retention time and mass fragmentation of the water extract to the authenticated standards, revealed multiple components of OCD20015-V009, including one benzoic acid (chlorogenic acid), 3 iridoids (catalpol, ajugol, and loganic acid), 5 flavonoids (epicatechin, calycosin-7-glucoside, isoquercitrin, calycosin, and formononetin), 7 triterpenoids (6α-hydroxypolyporenic acid C, dehydrotumulosic acid, poricoic acid A, polyporenic acid C, 3-epidehydrotumulosic acid, dehydropachymic acid, and pachymic acid), 19 triterpenoid saponins (platy saponin A, ginsenoside Re, ginsenoside Rg1, platycodin D2, platycodin D, ginsenoside Rf, ginsenoside Rb1, notoginsenoside R2, ginsenoside Rc, ginsenoside Ro, ginsenoside Rd, astragaloside II, isoastragalosides II, astragaloside II isomer, astragaloside I, isoastragaloside I, ginsenoside Rg5, astragaloside I isomer, and ginsenoside Rk2) (Figure 5 and Table 2). The antiviral effect of OCD20015-V009 on the IAV may be attributed to the effects of these compounds.

**TABLE 2:** The phytochemical profile of OCD20015-V009 by UHPLC-MS/MS.

No	Rt (min)	Calculated (m/z)	Estimated (m/z)	Adducts	Error (ppm)	Formula	MS/MS	Identifications
							Fragments (m/z)	
1	1.92	407.120	407.118	[M + COOH]^-^	−3.13	C_15_H_22_O_10_	199, 166	Catalpol^a^
2	4.51	393.140	393.139	[M + COOH]^-^	−2.64	C_15_H_24_O_9_	347, 149, 127	Ajugol^a^
3	4.73	375.130	375.129	[M-H]^-^	−2.44	C_16_H_24_O_10_	213, 169, 113	Loganic acid^a^
4	5.14	353.088	353.087	[M-H] ^-^	−2.62	C_16_H_18_O_9_	191	Chlorogenic Acid^a^
5	5.63	289.072	289.071	[M-H] ^-^	−2.47	C_15_H_14_O_6_	289, 245, 205	Epicatechin^a^
6	6.64	447.129	447.127	[M + H]^+^	−3.96	C_22_H_22_O_10_	283, 268	Calycosin-7-glucoside^a^
7	6.66	463.088	463.087	[M-H] ^-^	−2.51	C_21_H_20_O_12_	301	Isoquercitrin^a^
8	7.98	827.444	827.441	[M-H]^-^	−2.92	C_42_H_68_O_16_	—	Platy saponin A
9	8.80	991.548	991.546	[M + COOH]^-^	−2.63	C_48_H_82_O_18_	946, 475, 161	Ginsenoside Re^a^
10	8.80	845.490	845.488	[M + COOH]^-^	−2.66	C_42_H_72_O_14_	161, 101	Ginsenoside Rg1^a^
11	9.58	283.061	283.060	[M-H]^-^	−3.17	C_16_H_12_O_5_	268	Calycosin^a^
12	9.94	1385.623	1385.618	[M-H]^-^	−3.80	C_63_H_102_O_33_	843, 469	Platycodin D2^a^
13	10.03	1223.570	1223.566	[M-H]^-^	−3.82	C_57_H_92_O_28_	681, 541, 469	Platycodin D^a^
14	11.33	845.490	845.488	[M + COOH]^-^	−3.02	C_42_H_72_O_14_	799, 637	Ginsenoside Rf^a^
15	11.55	1153.601	1153.598	[M + COOH]^-^	−3.13	C_54_H_92_O_23_	1107, 945	Ginsenoside Rb1^a^
16	11.69	815.480	815.477	[M + COOH]^-^	−2.97	C_41_H_70_O_13_	637, 161	Notoginsenoside R2^a^
17	11.88	1123.591	1123.586	[M + COOH]^-^	−3.70	C_53_H_90_O_22_	1077, 945	Ginsenoside Rc^a^
18	12.10	955.491	955.487	[M-H]^-^	−3.59	C_48_H_76_O_19_	955, 793, 523	GinsenosideRo^a^
19	12.38	267.066	267.065	[M-H]^-^	−3.53	C_16_H_12_O_4_	252	Formononetin^a^
20	12.97	991.548	991.546	[M + COOH]^-^	−2.75	C_48_H_82_O_18_	621	Ginsenoside Rd^a^
21	13.71	871.470	871.467	[M + COOH]^-^	−2.81	C_43_H_70_O_15_	-	Astragaloside II
22	14.36	871.470	871.467	[M + COOH]^-^	−3.02	C_43_H_70_O_15_	-	Isoastragalosides II
23	14.96	871.470	871.467	[M + COOH]^-^	−3.16	C_43_H_70_O_15_	-	Astragaloside II isomer
24	15.60	913.480	913.478	[M + COOH]^-^	−2.96	C_45_H_72_O_16_	-	Astragaloside I
25	16.11	497.327	497.326	[M-H]^-^	−2.72	C_31_H_46_O_5_	419, 405, 403	6α-Hydroxypolyporenic acid C^a^
26	16.20	913.480	913.477	[M + COOH]^-^	−3.09	C_45_H_72_O_16_	-	Isoastragaloside I
27	16.55	767.494	767.490	[M + H] ^+^	−4.67	C_42_H_70_O_12_	443, 425, 407	Ginsenoside Rg5^a^
28	16.84	913.480	913.477	[M + COOH]^-^	−3.16	C_45_H_72_O_16_	-	Astragaloside I isomer
29	18.19	483.348	483.346	[M-H]^-^	−3.32	C_31_H_48_O_4_	437, 423, 405, 389	Dehydrotumulosic acid^a^
30	18.33	811.485	811.482	[M + COOH]^-^	−3.17	C_42_H_70_O_12_	765, 603	Ginsenoside Rk2^a^
31	18.57	497.327	497.326	[M-H]^-^	−3.03	C_31_H_46_O_5_	423, 379, 211	Poricoic acid A^a^
32	18.94	481.332	481.331	[M-H]^-^	−3.22	C_31_H_46_O_4_	435, 421, 311	Polyporenic acid C^a^
33	19.22	483.348	483.346	[M-H]^-^	−3.20	C_31_H_48_O_4_	437, 423, 337	3-Epidehydrotumulosic acid^a^
34	20.31	525.359	525.357	[M-H]^-^	−3.08	C_33_H_50_O_5_	465, 355	Dehydropachymic acid^a^
35	20.54	527.374	527.373	[M−H]^-^	−2.88	C_33_H_52_O_5_	527, 405	Pachymic acid^a^

a Compared to the retention time (Rt) and mass spectral data of reference standards.

### Viral Replication Inhibitory Effect of the Components Identified in OCD20015-V009

**FIGURE 6:**
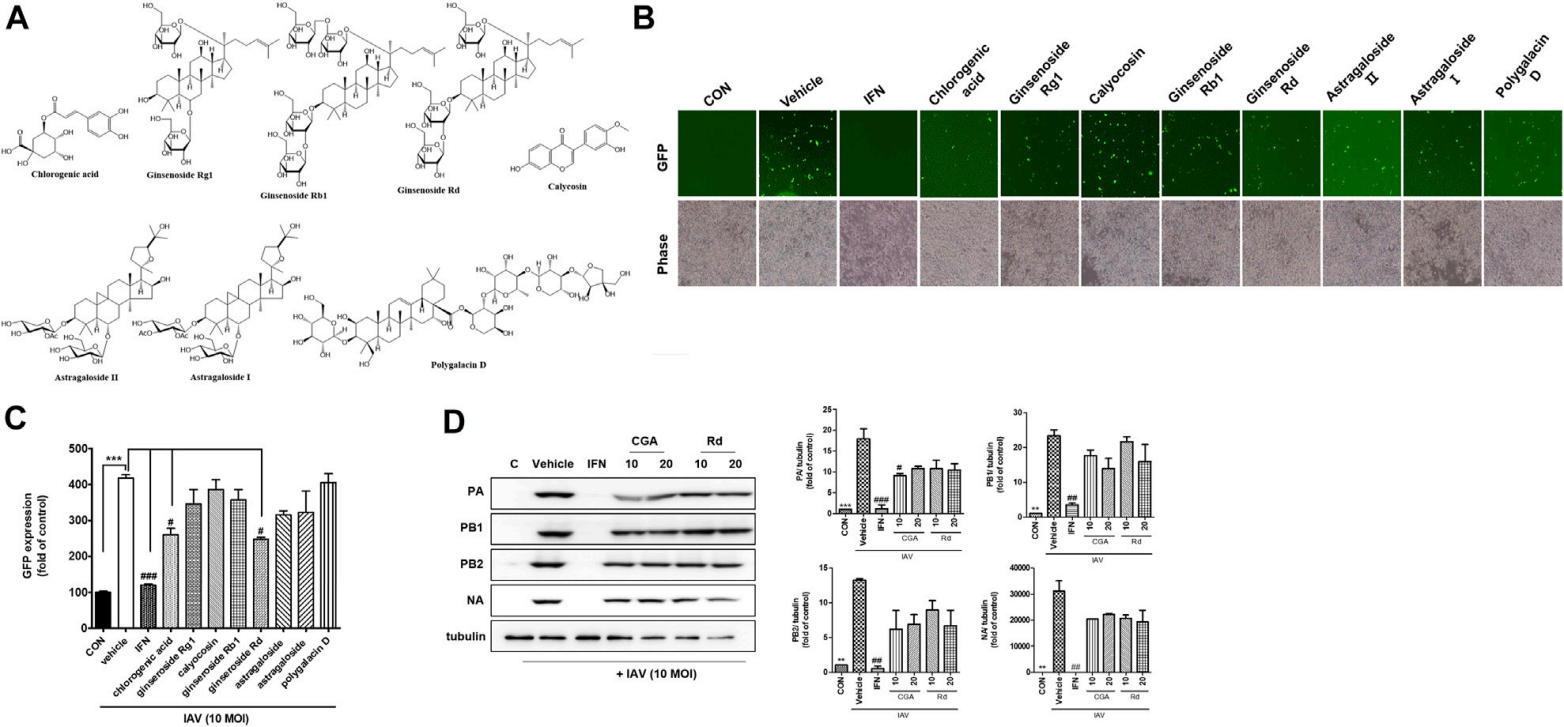
The antiviral effects of the eight major components identified in OCD20015-V009 on RAW 264.7 murine macrophages infected with influenza A virus (IAV). **(A)** The eight major components identified in OCD20015-V009: chlorogenic acid, ginsenoside Rg1, calycosin, ginsenoside Rb1, ginsenoside Rd, astragaloside II, astragaloside I, and polygalacin D. **(B)** RAW 264.7 cells were incubated with the medium alone, 10 μM of a major component of OCD20015-V009 or 1000 U/mL recombinant mouse interferon-β 12 h before infection with A/PR/8/34-GFP at the multiplicity of infection of 10. The images of GFP levels were obtained 24 h after virus infection. **(C)** The GFP levels and reduction in viral replication in RAW 264.7 cells pre-treated with the eight major components of OCD20015-V009 24 h after viral infection were assessed using flow cytometry. The bar graphs are plotted with the data from three experiments. ^***^ *p* < 0.001 compared with the control, ^#^ *p* < 0.05 and ^###^ *p* < 0.001 compared with the vehicle (IAV-infection). **(D)** The level of the IAV proteins in RAW 264.7 cells as assayed by Western blots using antibodies against various IAV proteins. RAW 264.7 cells were pre-treated with 10 and 20 μM chlorogenic acid or ginsenoside Rd, 1000 U/mL recombinant mouse interferon (IFN)-β, or the medium only (negative control) after viral adsorption. The levels of IAV proteins PA, PB1, PB2, and NA in the cell lysates were analyzed with Western blots; the level of tubulin was used as the internal control. Western blotting of viral expressions and then quantification using the ImageJ software. The data are presented as the mean ± SD (error bars) of three independent experiments. **p < 0.01 and ***p < 0.001 indicate a significant difference between control groups, #*p* < 0.05, ##*p* < 0.01 and ###*p* < 0.001 indicate a significant difference between the vehicle (IAV-infection).

Next, we investigated whether the eight major compounds in OCD20015-V009, chlorogenic acid, ginsenoside Rg1, calycosin, ginsenoside Rb1, ginsenoside Rd, astragaloside II, astragaloside I, and polygalacin D, inhibited H1NA influenza virus replication in RAW 264.7 cells by suppressing the production of the viral proteins. The GFP-expressing influenza virus A/PR/8/34-GFP was used to infect the RAW 264.7 cells. The level of GFP was lower in cells pre-treated with chlorogenic acid or ginsenoside Rd than that in the untreated cells (Figures 6A–C). Western blots showed that the levels of IAV proteins were suppressed in the RAW 264.7 cells pre-treated with chlorogenic acid or ginsenoside Rd compared to those in the untreated cells (Figure 6D).

## Conclusion

We additionally experimented on the co- and post-treatment antiviral activity of OCD20015-V009. However, the results demonstrated that OCD20015-V009 had no anti-influenza virus activity co- and post-treatment (Supplementary Figure S1). Therefore, these data indicated that OCD20015-V009 has anti-influenza A virus activity in the pre-treatment assays, but not in co- and post-treatment assays.

Here, we have discovered that OCD20015-V009 pre-treatment substantially reduces viral infection, based on the analysis of the infection of RAW 264.7 cells by A/PR/8/34-GFP. We have demonstrated that OCD20015-V009 pre-treatment increases the phosphorylation of type-I IFN-related proteins STAT1 and TBK-1 and the secretion of pro-inflammatory cytokines TNF-α and IL-6 in RAW 264.7 cells. Additionally, OCD20015-V009 pre-treatment increased the mRNA of the ISGs-related ISG 15, 20 and 56 and the IFN-β gene *in vitro*. Thus, these findings show that OCD20015-V009 has immunomodulatory and antiviral properties. This finding shows potential to develop prophylactic or therapeutic treatments against the IAV. In mice exposed to the H1N1 IAV, an OCD20015-V009 pre-treatment at 300 mg/kg decreased mortality by 20% compared with that in untreated animals. These data suggest that OCD20015-V009 stimulates an antiviral response in murine macrophages and mice, thus protecting against IAV infection. Additionally, we identified active compounds in OCD20015-V009, chlorogenic acid, or ginsenoside Rd, using UPLC-MS/MS and demonstrated that they exhibited the most significant antiviral effects.

Further investigations are warranted to elucidate the molecular mechanisms for the protective effects of OCD20015-V009 and its components, chlorogenic acid, and ginsenoside Rd, against influenza virus A infection. Based on the results, we propose that OCD20015-V009 and its components could be effective antiviral agents or vaccine adjuvants for influenza virus infection.

## Data Availability

The original contributions presented in the study are included in the article/Supplementary Material, further inquiries can be directed to the corresponding authors.

## References

[B1] Alymova I. V. Taylor G. Portner A. (2005). Neuraminidase Inhibitors as Antiviral Agents. Curr. Drug Targets Infect. Disord. 5, 401–409. 10.2174/156800505774912884 16535861

[B2] Berlanda Scorza F. Tsvetnitsky V. Donnelly J. J. (2016). Universal Influenza Vaccines: Shifting to Better Vaccines. Vaccine 34, 2926–2933. 10.1016/j.vaccine.2016.03.085 27038130 PMC4899887

[B3] Chen X. Liu S. Goraya M. U. Maarouf M. Huang S. Chen J. L. (2018). Host Immune Response to Influenza A Virus Infection. Front. Immunol. 9, 320. 10.3389/fimmu.2018.00320 29556226 PMC5845129

[B4] Choi J. G. Jin Y. H. Kim J. H. Oh T. W. Yim N. H. Cho W. K. (2016). *In Vitro* Anti-viral Activity of Psoraleae Semen Water Extract against Influenza A Viruses. Front. Pharmacol. 7, 460. 10.3389/fphar.2016.00460 27965579 PMC5127801

[B5] Choi J. G. Jin Y. H. Lee H. Oh T. W. Yim N. H. Cho W. K. (2017a). Protective Effect of Panax Notoginseng Root Water Extract against Influenza A Virus Infection by Enhancing Antiviral Interferon-Mediated Immune Responses and Natural Killer Cell Activity. Front. Immunol. 8, 1542. 10.3389/fimmu.2017.01542 29181006 PMC5693858

[B6] Choi J. G. Kim Y. S. Kim J. H. Chung H. S. (2019). Antiviral Activity of Ethanol Extract of Geranii Herba and its Components against Influenza Viruses via Neuraminidase Inhibition. Sci. Rep. 9, 12132. 10.1038/s41598-019-48430-8 31431635 PMC6702199

[B7] Choi J. G. Lee H. Hwang Y. H. Lee J. S. Cho W. K. Ma J. Y. (2017b). Eupatorium Fortunei and its Components Increase Antiviral Immune Responses against RNA Viruses. Front. Pharmacol. 8, 511. 10.3389/fphar.2017.00511 28824435 PMC5541272

[B8] Clayville L. R. (2011). Influenza Update: a Review of Currently Available Vaccines. P T 36, 659–684. 22346299 PMC3278149

[B9] D'alessandro S. Corbett Y. Parapini S. Perego F. Cavicchini L. Signorini L. (2019). Malaria Pigment Accelerates MTT - Formazan Exocytosis in Human Endothelial Cells. Parasitology 146, 399–406. 10.1017/S0031182018001579 30269694

[B10] Degoot A. M. Adabor E. S. Chirove F. Ndifon W. (2019). Predicting Antigenicity of Influenza A Viruses Using Biophysical Ideas. Sci. Rep. 9, 10218. 10.1038/s41598-019-46740-5 31308446 PMC6629677

[B11] Gu Y. Hsu A. C. Pang Z. Pan H. Zuo X. Wang G. (2019). Role of the Innate Cytokine Storm Induced by the Influenza A Virus. Viral Immunol. 32, 244–251. 10.1089/vim.2019.0032 31188076

[B12] Hayden F. G. Shindo N. (2019). Influenza Virus Polymerase Inhibitors in Clinical Development. Curr. Opin. Infect. Dis. 32, 176–186. 10.1097/QCO.0000000000000532 30724789 PMC6416007

[B13] Hussain M. Galvin H. D. Haw T. Y. Nutsford A. N. Husain M. (2017). Drug Resistance in Influenza A Virus: the Epidemiology and Management. Infect. Drug Resist. 10, 121–134. 10.2147/IDR.S105473 28458567 PMC5404498

[B14] Hwang Y. H. Jang S. A. Kim T. Ha H. (2019). Forsythia Suspensa Protects against Bone Loss in Ovariectomized Mice. Nutrients 11, 1831. 10.3390/nu11081831 PMC6722587 31398803

[B15] Hwang Y. H. Jang S. A. Lee A. Kim T. Ha H. (2020). Poria Cocos Ameliorates Bone Loss in Ovariectomized Mice and Inhibits Osteoclastogenesis *In Vitro* . Nutrients 12, 1383. 10.3390/nu12051383 PMC7284350 32408635

[B16] Jewell N. A. Vaghefi N. Mertz S. E. Akter P. Peebles R. S. Jr. Bakaletz L. O. (2007). Differential Type I Interferon Induction by Respiratory Syncytial Virus and Influenza a Virus *In Vivo* . J. Virol. 81, 9790–9800. 10.1128/JVI.00530-07 17626092 PMC2045394

[B17] Kim Y. S. Li W. Kim J. H. Chung H. S. Choi J. G. (2020). Anti-Influenza Activity of an Ethyl Acetate Fraction of a Rhus Verniciflua Ethanol Extract by Neuraminidase Inhibition. Oxid Med. Cel Longev 2020, 8824934. 10.1155/2020/8824934 PMC7661131 33204399

[B18] Koerner I. Kochs G. Kalinke U. Weiss S. Staeheli P. (2007). Protective Role of Beta Interferon in Host Defense against Influenza A Virus. J. Virol. 81, 2025–2030. 10.1128/JVI.01718-06 17151098 PMC1797552

[B19] Kopitar-Jerala N. (2017). The Role of Interferons in Inflammation and Inflammasome Activation. Front. Immunol. 8, 873. 10.3389/fimmu.2017.00873 28791024 PMC5525294

[B20] Kumagai Y. Takeuchi O. Kato H. Kumar H. Matsui K. Morii E. (2007). Alveolar Macrophages Are the Primary Interferon-Alpha Producer in Pulmonary Infection with RNA Viruses. Immunity 27, 240–252. 10.1016/j.immuni.2007.07.013 17723216

[B21] Lam T. T. Wang J. Shen Y. Zhou B. Duan L. Cheung C. L. (2013). The Genesis and Source of the H7N9 Influenza Viruses Causing Human Infections in China. Nature 502, 241–244. 10.1038/nature12515 23965623 PMC3801098

[B22] Lee D. Y. Choi B.-R. Lee J. W. Um Y. Yoon D. Kim H.-G. (2019). Simultaneous Determination of Various Platycosides in Four Platycodon Grandiflorum Cultivars by UPLC-QTOF/MS. Appl. Biol. Chem. 62, 47. 10.1186/s13765-019-0457-x

[B23] Mosmann T. (1983). Rapid Colorimetric Assay for Cellular Growth and Survival: Application to Proliferation and Cytotoxicity Assays. J. Immunol. Methods 65, 55–63. 10.1016/0022-1759(83)90303-4 6606682

[B24] Mousa H. A. (2017). Prevention and Treatment of Influenza, Influenza-like Illness, and Common Cold by Herbal, Complementary, and Natural Therapies. J. Evid. Based Complement. Altern Med. 22, 166–174. 10.1177/2156587216641831 PMC5871211 27055821

[B25] Pardi N. Weissman D. (2020). Development of Vaccines and Antivirals for Combating Viral Pandemics. Nat. Biomed. Eng. 4, 1128–1133. 10.1038/s41551-020-00658-w 33293724 PMC8336060

[B26] Santoro V. Parisi V. D'ambola M. Sinisgalli C. Monne M. Milella L. (2020). Chemical Profiling of Astragalus Membranaceus Roots (Fish.) Bunge Herbal Preparation and Evaluation of its Bioactivity. Nat. Product. Commun. 15, 1–11. 10.1177/1934578x20924152

[B27] Takeda M. Pekosz A. Shuck K. Pinto L. H. Lamb R. A. (2002). Influenza a Virus M2 Ion Channel Activity Is Essential for Efficient Replication in Tissue Culture. J. Virol. 76, 1391–1399. 10.1128/jvi.76.3.1391-1399.2002 11773413 PMC135863

[B28] Talactac M. R. Chowdhury M. Y. Park M. E. Weeratunga P. Kim T. H. Cho W. K. (2015). Antiviral Effects of Novel Herbal Medicine KIOM-C, on Diverse Viruses. PLoS One 10, e0125357. 10.1371/journal.pone.0125357 25942440 PMC4420246

[B29] Teijaro J. R. (2016). Type I Interferons in Viral Control and Immune Regulation. Curr. Opin. Virol. 16, 31–40. 10.1016/j.coviro.2016.01.001 26812607 PMC4821698

[B30] Trinh T. A. Park J. Oh J. H. Park J. S. Lee D. Kim C. E. (2020). Effect of Herbal Formulation on Immune Response Enhancement in RAW 264.7 Macrophages. Biomolecules 10, 424. 10.3390/biom10030424 PMC7175197 32182890

[B31] Vicidomini C. Roviello V. Roviello G. N. (2021b). Molecular Basis of the Therapeutical Potential of Clove (Syzygium Aromaticum L.) and Clues to its Anti-COVID-19 Utility. Molecules 26, 1880. 10.3390/molecules26071880 33810416 PMC8036487

[B32] Vicidomini C. Roviello V. Roviello G. N. (2021a). In Silico Investigation on the Interaction of Chiral Phytochemicals from Opuntia Ficus-Indica with SARS-CoV-2 Mpro. Symmetry 13, 1041. 10.3390/sym13061041

[B33] Wu W. Metcalf J. P. (2020). The Role of Type I IFNs in Influenza: Antiviral Superheroes or Immunopathogenic Villains. J. Innate Immun. 12, 437–447. 10.1159/000508379 32564033 PMC7747089

[B34] Yin R. Tran V. H. Zhou X. Zheng J. Kwoh C. K. (2018). Predicting Antigenic Variants of H1N1 Influenza Virus Based on Epidemics and Pandemics Using a Stacking Model. PLoS One 13, e0207777. 10.1371/journal.pone.0207777 30576319 PMC6303045

[B35] Zhang J. Hu Y. Musharrafieh R. Yin H. Wang J. (2019). Focusing on the Influenza Virus Polymerase Complex: Recent Progress in Drug Discovery and Assay Development. Curr. Med. Chem. 26, 2243–2263. 10.2174/0929867325666180706112940 29984646 PMC6426683

