# Supplementary material for: A Herbal Mixture Formula of OCD20015-V009 Prophylactic Administration to Enhance Interferon-Mediated Antiviral Activity Against Influenza A Virus
Source: Front Pharmacol. 2021 Nov 24;12:764297. doi: 10.3389/fphar.2021.764297 (PMC8651992; doi:10.3389/fphar.2021.764297)
Supplement: Supplementary file 1 [file Image1.pdf]

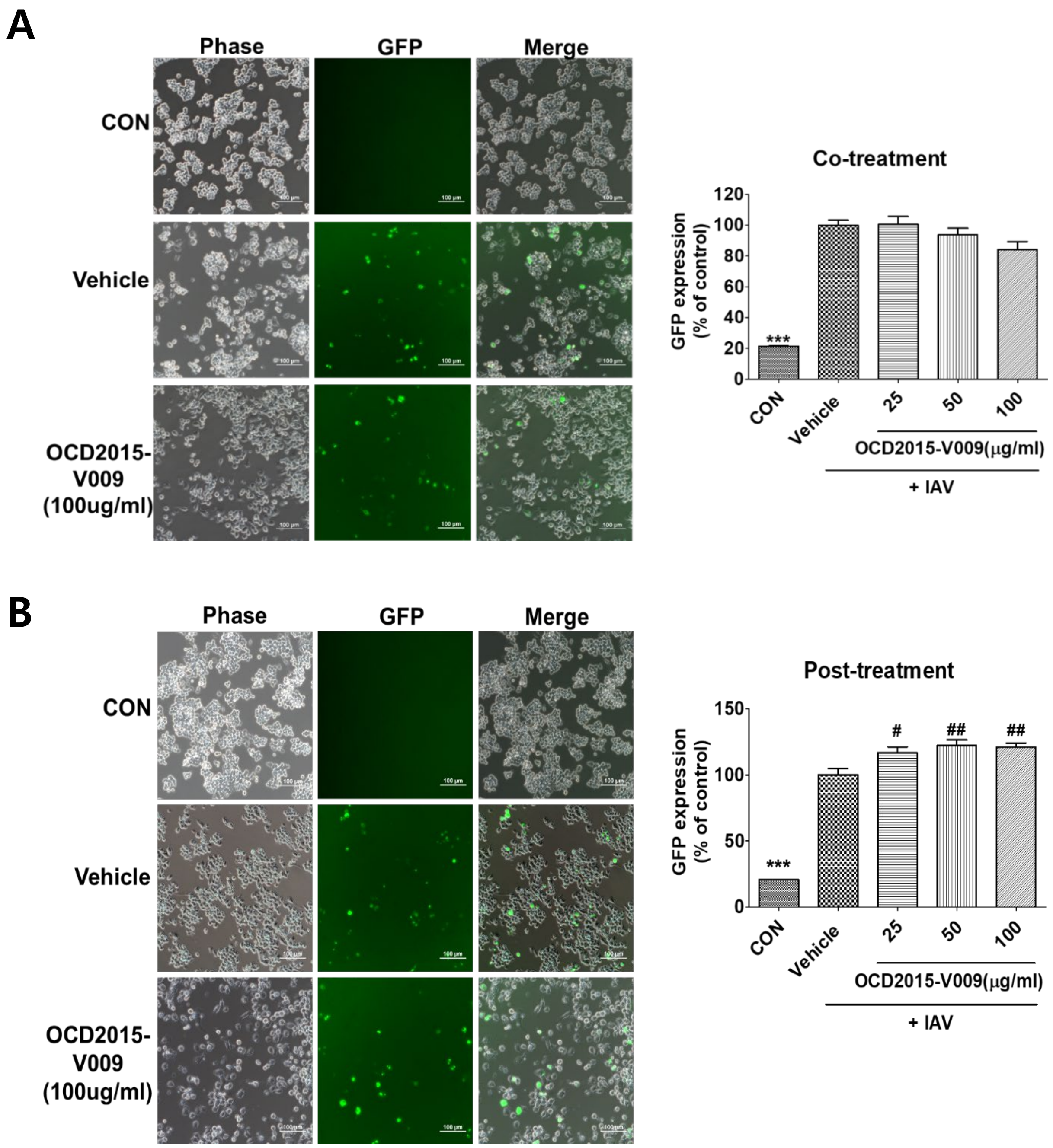

Figure S1. The antiviral of OCD20015-V009 co- and post-treatment on the influenza A virus infection in RAW264.7 murine macrophages. (A) For the co-treatment assay, the indicated OCD20015-V009 concentrations were mixed with 10 MOI A/PR/8/34-GFP and the mixture was incubated at 4°C for 1 h. The cells were were infected with the virus mixture at 37°C for 2 h. Afterward, the virus was removed and replaced by complete DMEM. The cultures were incubated for the indicated time at 37°C and 5% CO2. (B) For the post-treatment assay, the RAW 264.7 cells were infected with 10 MOI PR/8/34-GFP at 37°C for 2 h. Afterward, the virus

was removed, and cells were treated with indicated concentrations of OCD20015-V009 in complete DMEM for the indicated time at 37°C and 5% CO_2. The GFP levels were measured at 24 h post-infection (hpi) at 200 × magnification under a fluorescence microscope (Nikon, Japan) or using flow cytometry (CytoFLEX, Beckman, USA).
